# Short-term combined treatment with liraglutide and metformin leads to significant weight loss in obese women with polycystic ovary syndrome and previous poor response to metformin

**DOI:** 10.1530/EJE-13-0797

**Published:** 2014-03

**Authors:** Mojca Jensterle Sever, Tomaz Kocjan, Marija Pfeifer, Nika Aleksandra Kravos, Andrej Janez

**Affiliations:** Department of Endocrinology, Diabetes and Metabolic DiseasesUniversity Medical Center LjubljanaZaloska 7, 1525 LjubljanaSlovenia

## Abstract

**Objective:**

The effect of metformin on weight reduction in polycystic ovary syndrome (PCOS) is often unsatisfactory. In this study, we investigated the potential add-on effect of treatment with the glucagon-like peptide-1 receptor agonist liraglutide on weight loss in obese nondiabetic women with PCOS who had lost <5% body weight during pretreatment with metformin.

**Methods:**

A total of 40 obese women with PCOS, who had been pretreated with metformin for at least 6 months, participated in a 12-week open-label, prospective study. They were randomized to one of three treatment arms: metformin (MET) arm 1000 mg BID, liraglutide (LIRA) arm 1.2 mg QD s.c., or combined MET 1000 mg BID and LIRA (COMBI) 1.2 mg QD s.c. Lifestyle intervention was not actively promoted. The primary outcome was change in body weight.

**Results:**

Thirty six patients (aged 31.3±7.1 years, BMI 37.1±4.6 kg/m^2^) completed the study: 14 on MET, 11 on LIRA, and 11 on combined treatment. COMBI therapy was superior to LIRA and MET monotherapy in reducing weight, BMI, and waist circumference. Subjects treated with COMBI lost on average 6.5±2.8 kg compared with a 3.8±3.7 kg loss in the LIRA group and a 1.2±1.4 kg loss in the MET group (*P*<0.001). The extent of weight loss was stratified: a total of 38% of subjects were high responders who lost ≥5% body weight, 22% of them in the COMBI arm compared with 16 and 0% in the LIRA and MET arm respectively. BMI decreased by 2.4±1.0 in the COMBI arm compared with 1.3±1.3 in LIRA and 0.5±0.5 in the MET arm (*P*<0.001). Waist circumference also decreased by 5.5±3.8 cm in the COMBI arm compared with 3.2±2.9 cm in LIRA and 1.6±2.9 cm in the MET arm (*P*=0.029). Subjects treated with liraglutide experienced more nausea than those treated with metformin, but severity of nausea decreased over time and did not correlate with weight loss.

**Conclusions:**

Short-term combined treatment with liraglutide and metformin was associated with significant weight loss and decrease in waist circumference in obese women with PCOS who had previously been poor responders regarding weight reduction on metformin monotherapy.

## Introduction

Metformin is an established treatment of polycystic ovary syndrome (PCOS). It appears to have multiple favorable effects on menstrual disorders, anovulation, hyperandrogenism, and, particularly, on metabolic and cardiovascular abnormalities in this population (1, 2, 3, 4). It is also useful in the management of anovulatory infertility in women with PCOS who have a BMI within the normal range. In addition, its administration reduces the incidence and severity of ovarian hyperstimulation syndrome during IVF in women with PCOS who are at high risk for this syndrome. Moreover, the combination of metformin with clomiphene appears to be the best treatment choice in clomiphene-resistant women and should precede the administration of gonadotropins (5). However, its effect on weight reduction as an adjuvant to lifestyle modification is often unsatisfactory (6, 7). Recognizing that weight loss substantially improves reproductive and metabolic profile of obese patients with PCOS, an alternative approach in poor responders regarding weight reduction should be of major importance in clinical intervention.

Glucagon-like peptide-1 (GLP1) analogs have been recently introduced as a new treatment for patients with type 2 diabetes mellitus. In addition to improvement in glucose homeostasis, they have also been shown to reduce body weight. The effect on body weight appears to be due to reduction in food intake, mainly by a direct hypothalamic effect of the hormone. It also delays gastric emptying partly due to a central action mediated via the autonomous nervous system. One of the side effects of GLP1 receptor agonist, nausea, could contribute to the weight reducing effect; however, weight loss has also been observed separately when analyzing patients who did not report nausea (8, 9). The published studies provide convincing evidence that GLP1 receptor agonist when given to obese patients with or without diabetes results in clinically relevant progressive and sustained weight loss (10). There are very limited data on weight loss in patients with PCOS treated with these agents. Until now, only one study has addressed GLP1 agonist treatment in PCOS, providing evidence that combined treatment with short-acting GLP1 receptor agonist exenatide and metformin was superior to exenatide and metformin monotherapies in improving menstrual cyclicity, ovulation rate, androgens, insulin sensitivity measures, and weight loss (11).

Liraglutide is a long-acting GLP1 analog, with 97% linear amino acid sequence homology to human GLP1 and a half-life of 13 h making it suitable for once-daily s.c. administration (12). It has been developed as an adjunct to lifestyle therapy, and in combination with oral antidiabetic drugs, for the treatment of type 2 diabetes mellitus. In PCOS, metformin has become an established treatment. The potential add-on effect of treatment with liraglutide has not been evaluated in this population yet. In this study, we report the results of a 12-week open-label, randomized, prospective study designed to investigate the effect of liraglutide on weight loss in obese nondiabetic women with PCOS who had lost <5% body weight during 6-month pretreatment with metformin.

## Subjects and methods

Between November 2011 and May 2012, 40 women were recruited from the outpatients Department of Endocrinology, Diabetes and Metabolic Diseases, University Medical Center Ljubljana. These women were screened out of 215 women with PCOS that underwent a routine follow-up and were previously diagnosed to have PCOS by the National Institute of Child Health and Human Development (NICHD) criteria (13). They were initially put on metformin because they had metabolic disturbances and/or anovulation. They were eligible for enrolment if they were more than 18 years old, premenopausal, obese (BMI ≥30 kg/m^2^), had been taking metformin 2000 mg for at least 6 months, and had lost <5% of body weight with average weight change +1.2±4.7 kg (mean±s.d.) in the last 6 months. Exclusion criteria were known type 1 or type 2 diabetes mellitus; history of carcinoma; personal or family history of *MEN2* (*RET*); significant cardiovascular, kidney, or liver disease; and the use of medications other than metformin known or suspected to affect reproductive or metabolic functions, or statins, within 90 days before study entry. Clinical hyperandrogenism defined by the presence of hirsutism was graded using the Ferriman–Gallwey scoring system with scores of ≥8 accepted as abnormal, persistence of acne during the third decade of life or later, or the presence of androgenic alopecia. Because these variables were not our outcomes and were not expected to change during the short period of observation, no attempts were made to quantify the severity of hirsutism, acne, or alopecia. Hyperandrogenemia was defined as a total or free testosterone, androstenedione, and/or DHEAS level above the 95th percentile of normal population values (values in the healthy matched population are presented in the legend of Table 1). Menstrual dysfunction was defined by more than six cycles with a length of more than 35 days (oligomenorrhea), and/or when the patient had not had any menstrual bleeding for three consecutive months (amenorrhea) during the previous year. All patients had normal serum prolactin concentrations and thyroid function tests. Possible Cushing's syndrome was excluded when clinically indicated. If only DHEAS level was elevated or when basic 17α-hydroxy-progesterone was >6.1 nmol/l (2 ng/ml), the determination of 17α-hydroxy-progesterone after stimulation with Synacthen (0.25 mg/ml; Novartis Pharma S.A.) was carried out to rule out nonclassic congenital adrenal hyperplasia (14). All subjects were informed of the study aims and provided written consent before entering the study, which was conducted in accordance with the Declaration of Helsinki and approved by the National Ethical Committee. The study is registered with clinicaltrials.gov identifier, NCT01911468.

### Experimental protocol

Fourty women with PCOS, who were obese and pretreated with metformin, participated in a 12-week open-label prospective, randomized, outpatient clinical study. They were randomized to one of three treatment arms: metformin (MET) 1000 mg BID, liraglutide (LIRA) 1.2 mg QD s.c., or combined MET 1000 mg BID, and LIRA 1.2 mg QD s.c. (COMBI). Lifestyle intervention was not actively promoted. As a method of randomization, the RAND program in Excel was used. In the LIRA group, metformin was discontinued and patients were switched to liraglutide at a dose of 0.6 mg injected s.c. once a day and increased to 1.2 mg/day after 1 week. In the COMBI group, liraglutide was added to MET on the same dosage schedule. The primary outcome was change in anthropometric measures of obesity. Prespecified secondary outcomes included endocrine and metabolic changes.

At baseline, after 4 and 8 weeks, and at study endpoint, all patients underwent standard anthropometric measurements: height, weight, waist circumference, and blood pressure (BP). Waist circumference was measured in a standing position midway between the lower costal margin and the iliac crest. BMI was calculated as the weight in kilograms divided by square of height in meters. Furthermore, measurement of whole-body composition on a Hologic dual energy X-ray absorptiometry (DXA) was carried out in all subjects at baseline and at study endpoint as described previously (15). We assumed that the different phases of the menstrual cycle had little or no effect on body weight and body composition (16). A fasting blood was drawn for determination of glucose, insulin, luteinizing hormone (LH), follicle-stimulating hormone (FSH), androstenedione, DHEAS, and total and free testosterone followed by a standard 75 g oral glucose tolerance test (OGTT). Safety parameters (complete blood count, markers of hepatic and renal functions, and serum electrolytes) were assessed before and after 12 weeks of study treatment. All blood samples were centrifuged and the separated sera were kept frozen at −80 °C until the time of the assay. Impaired glucose tolerance (IGT) was identified by 2 h glucose levels between 7.8 and 11.0 mmol/l, as defined by the American Diabetes Association criteria (17). As many of the patients were oligoamenorrhoic during the previous year, the assessment of the subjects was not based on any specific stage of the menstrual cycle. Pregnancy was excluded by measuring β-human chorionic gonadotropin.

Women were advised to strictly use barrier contraception. All patients were provided with glucose-monitoring devices, medication supplies, and educated on their use. They were instructed to measure blood glucose levels at any signs and symptoms suggesting low blood glucose. According to American Diabetes Association criteria, hypoglycemia was defined as symptoms suggestive of low blood glucose confirmed by self-monitored blood glucose measurement below 3.9 mmol/l (17). They were also instructed to report any side effects during the treatment. An energy restricted (500–800 kcal/day reduction; increased consumption of fiber, whole-grain breads, cereals, fruits, and vegetables) along with at least 30 min of moderate intensity physical activity daily had been recommended to all women when the diagnosis of PCOS had been confirmed and metformin had been initially prescribed. At the beginning of the study, lifestyle intervention was not again actively promoted.

### Assays

Glucose levels were determined using a standard glucose oxidase method (Beckman Coulter Glucose Analyzer, Beckman Coulter, Inc., CA, USA). LH and FSH were determined using an immunometric assay (Diagnostic Products Corporation, Los Angeles, CA, USA). Androstenedione and DHEAS were measured by specific double-antibody RIA using ^125^I-labeled hormones (Diagnostic Systems Laboratories, Webster, TX, USA). Total and free testosterone levels were measured by coated-tube RIA (DiaSorin, S.p.A. (Salluggia, Italy) and Diagnostic Products Corporation respectively). Insulin was determined by IRMA (Biosource Europe S.A., Nivelles, Belgium). Intraassay variations ranged from 1.6 to 6.3%, and interassay variations ranged from 5.8 to 9.6% for the applied methods. Pre- and posttreatment samples from each patient were assayed in the same assay run.

### Determination of metabolic syndrome

According to the International Diabetes Federation definition, the metabolic syndrome in women is defined as central obesity (defined as waist circumference >80 cm), plus any two of the following four factors: increased triglycerides ≥1.7 mmol/l, reduced HDL cholesterol <1.29 mmol/l, increased BP (systolic BP ≥130 mmHg or diastolic BP ≥85 mmHg), increased fasting plasma glucose (FPG) ≥5.6 mmol/l (17).

### Determination of insulin resistance

Homeostasis model assessment of insulin resistance (HOMA_IR_) score calculation was applied as a measure for IR. The estimate of IR by HOMA_IR_ score was calculated with the following formula: fasting serum insulin (mU/l)×FPG (mmol/l)/22.5 (18). HOMA_IR_ score values of 2.0 were considered as a cut off point for IR as published previously (19).

### Assessment of human body composition by DXA

Whole-body composition was assessed by a DXA (Discovery A; Hologic, Waltham, MA, USA) with the software provided by the manufacturer (QDR for Windows, version 12.5). The instrument generates values for whole-body fat mass, lean body mass, and bone mineral content.

### Statistical analysis

Results are presented as mean±s.d. Normal data distribution was checked with the Shapiro–Wilk test. For the normally distributed data, we used the one-way ANOVA with the *post-hoc* Bonferroni's test for a precise comparison of the differences amongst the arms and the Kruskal–Wallis test with the *post-hoc* Bonferroni's test for non-normal data distribution; to determine the differences between individual arms (COMBI, LIRA, and MET) the Mann–Whitney *U* test was used. The differences between time intervals (baseline, 3 months) were analyzed with the multilevel analysis (linear mixed model), by which method the variance of the dependable variables is analyzed on multiple hierarchical levels (between different arms – COMBI, LIRA, and MET). *P* value of <0.05 was considered statistically significant. All statistical analyses were carried out using the SPSS 17.0 Statistical Software Package.

## Results

### Baseline results

Thirty-six patients (11 on LIRA, 14 on MET, and 11 on COMBI) finished the study according to the protocol and were included in the analysis. Two patients were excluded from the LIRA group due to severe nausea and one from the COMBI group due to acute cholangitis caused by gallstones. One patient on COMBI was lost due to protocol violation. Baseline characteristics of the study population are provided in Table 1. As expected because of randomization, there were no significant differences at baseline in any of the parameters between the treatment groups.

### Weight change

**Figure 1:**
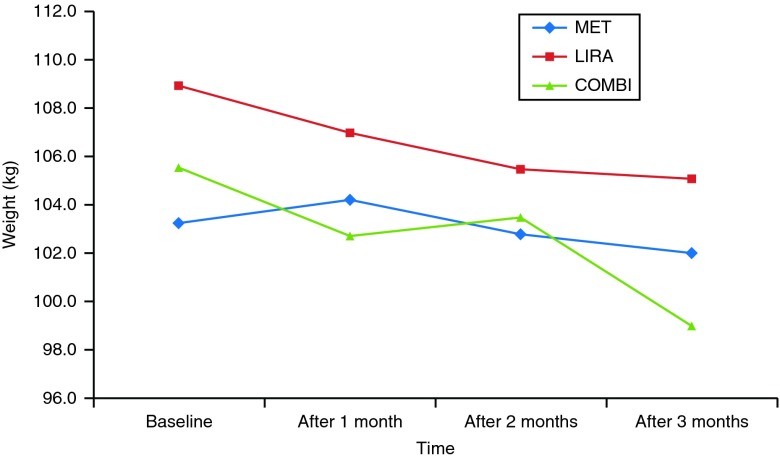
Weight change per arm during 3-month follow-up.

At 3 months, body mass significantly decreased in all treatment arms (*P*<0.001 for the treatment/time effect). Subjects treated with COMBI lost on average 6.5±2.8 kg compared with a 3.8±3.7 kg loss in the LIRA group and 1.2±1.4 kg loss in the MET group (*P*<0.001 for the differences between the COMBI and MET therapy arms). A total of 38% of subjects were high responders who lost ≥5% body weight in 3 months, 22% of them in the COMBI arm compared with 16% in the LIRA arm. No subject in the MET arm achieved this degree of weight loss. Timeline of weight change per arm from baseline to 3 months on the therapy is presented in Fig. 1.

BMI decreased for 2.4±1.0 kg/m^2^ in the COMBI arm compared with 1.3±1.3 kg/m^2^ in LIRA and 0.5±0.5 kg/m^2^ in the MET arm (*P*<0.001 for the effect of time). The analysis showed statistically significant differences between the COMBI and MET therapy arms (*P*<0.001).

Comparable results were found for reduction in body waist circumference (*P*=0.049 for the treatment/time effect). The maximum waist circumference reduction was noted in patients on COMBI therapy followed by the LIRA and MET therapy arm patients (with treatment differences 5.5±3.8, 3.2±2.9, and 1.6±2.9 cm, respectively, *P*=0.050 for the COMBI vs MET). All treatment interventions resulted in a nonsignificant reduction in visceral fat, as assessed by DXA. At 3 months, an average visceral adipose tissue area in all groups decreased below 160 cm^2^, a threshold that is associated with high risk for cardiovascular disease in women (20).

### Metabolic changes

Twelve women (33% of all patients: six in MET, four in LIRA, and two in the COMBI group) had IGT at the beginning of the study. Five (42%) of these women had normal glucose tolerance after 3 months of treatment (one on MET, three on LIRA, and one on the COMBI). Six women in the MET group, four in COMBI, and seven in the LIRA group had metabolic syndrome at baseline. After 3 months of treatment, the metabolic syndrome was resolved in three out of seven patients in the LIRA group and persisted in all six women in MET and in three in the COMBI group. At baseline, nine patients in MET, five in COMBI, and five in the LIRA group were insulin resistant according to their HOMA_IR_ score (>2). After 12 weeks of treatment, HOMA_IR_ score >2 was found in six patients in the MET group, six in COMBI, and five in the LIRA group. Overall, HOMA_IR_ score values tended to be reduced after all three interventions, but did not significantly decrease in any group. Fasting glucose, insulin, and insulin during OGTT did not consistently improve either. However, patients in the COMBI arm proved the most successful at reducing glucose value after 120 min during OGTT (*P*=0.006 for COMBI vs MET).

### Endocrine changes

There was a significant differential effect of COMBI compared with LIRA and MET with respect to androstenedione levels, with COMBI leading to a significant decrease in androstenedione level (−2.2±3.7 nmol/l) and LIRA and MET leading to a significant increase (1.9±4.2 and 0.8±1.70 nmol/l respectively). No statistically significant changes were found, neither when comparing other hormones irrespective of the therapeutic arm nor when analyzing it separately by therapeutic arm (Table 1).

### Changes in menstrual pattern

Menstruation frequency was also included as a secondary endpoint. No statistically significant changes were found, neither over time nor when analyzing it separately by therapeutic arm.

### Adverse events

The most frequent adverse events in patients included in the analysis were mild or moderate and gastrointestinal (GI) in nature. They were more frequent in LIRA and COMBI treatment arms than in MET. In the MET group, one (1/14) subject had temporary mild GI problems (nausea and diarrhea) and another one had hypoglycemic events. Adverse events associated with LIRA were nausea (6/11), diarrhea (6/11), headache (3/11), and rash at the injection site during the first week (1/11). Two patients in the LIRA group reported minor hypoglycemic events. Patients in the COMBI group had nausea (6/11) and diarrhea (6/11), headache (3/11), insomnia (2/11), and three of them had minor hypoglycemic events. Gradual dose titration reduced the GI side effects associated with liraglutide. The need for daily s.c. injections of liraglutide was generally not less appealing than oral application of metformin twice daily.

## Discussion

Recent clinical trials in obese subjects without diabetes have indicated that GLP1 receptor agonist therapy does decrease body weight up to 5–10% (21). The potentially beneficial role of long-acting GLP1 receptor agonist liraglutide alone or in combination with metformin on weight reduction in PCOS has not yet been evaluated. To our knowledge, this is the first study to date demonstrating that short-term 12 weeks' combined treatment with liraglutide and metformin was associated with significant weight loss in obese women with PCOS who had previously been poor responders regarding weight reduction on metformin monotherapy.

We observed that women who took the combination of drugs lost weight, on average 6.5±2.8 kg compared with a 3.8±3.7 kg loss in the LIRA group and a 1.2±1.4 kg loss in the MET group. Thirty-eight percent of participants lost a significant amount of weight, defined as 5% or more of their initial body weight; 22% of those were on the COMBI treatment compared with 16% on LIRA. No subject in the MET group achieved this amount of weight loss although all treatments caused progressive and sustained weight loss. Although we did not identify any baseline characteristics that might have predicted the degree of response to liraglutide treatment, a robust weight loss in a subset of individuals was apparent after 4 weeks of treatment and continued to the end of the study. The greatest reduction in body weight was achieved during the 8th to the 12th week of the observation period in the COMBI treatment arm. In terms of BMI and waist circumference, the COMBI treatment group achieved greater improvements than either of the single-medication groups. For both of these measurements, LIRA alone outperformed MET alone. However, the observed weight and waist circumference reductions have not yet been accompanied by significant improvement in body composition as measured by DXA.

It seems that the weight reduction achieved in our study is of comparable magnitude to previous trials using GLP1 receptor agonist as an anti-obesity treatment in obese subjects without diabetes, but it was achieved in a shorter period of time. In a study by Rosenstock *et al*. (22), obese patients were randomized to receive exenatide or placebo along with lifestyle intervention for 24 weeks. Exenatide-treated subjects lost 5.1 vs 1.6 kg with placebo. In another 20-week trial, obese subjects with a BMI range from 30 to 40 kg/m^2^ were randomized to liraglutide, placebo, or orlistat. Mean weight loss with 1.2 mg liraglutide was 4.8 kg compared with 2.8 kg with placebo and 4.1 kg with orlistat (23). Orlistat along with an energy-restricted diet was also prescribed in a recent 24-week study designed to assess the effect of weight loss on serum adipokine levels in PCOS. A significant reduction in BMI was observed in patients with PCOS (34.9 at baseline compared with 30.4 kg/m^2^ after 24 weeks) and controls (34.9 vs 29.9 kg/m^2^) (24). According to these reports, weight reduction with GLP1 agonist and orlistat seems to be comparable. However, the potential advantage of GLP1 agonists over orlistat in the obese PCOS population is the expected additional improvement of glucose–insulin homeostasis not being observed with orlistat (25).

To date, only one study has addressed the possible use of GLP1 receptor agonist in women with PCOS. However, as opposed to the long-acting GLP1 analog, liraglutide, used in our study, they used short-acting GLP-1 analog, exenatide, in treatment-naive overweight patients (BMI >27 kg/m^2^) with PCOS for 24 weeks (11). Their primary outcome was change in menstrual frequency, whereas change in body weight was prespecified as the secondary outcome. After 24 weeks, the combined group treated with exenatide and metformin had a mean weight loss of 6±0.5 kg, the exenatide group had a mean weight loss of 3.2±0.1 kg, and the metformin group had a mean loss of 1.6±0.2 kg. The effect on body weight in our study was comparable with those results, but achieved in half of that time. In agreement with our findings, combined treatment was superior to both monotherapies, which suggested a possible additive effect of metformin to GLP1 receptor agonist. Metformin may exert its additive beneficial action in part through the modulation of the incretin axis (26) by the stimulatory effect of GLP1, leading to enhancement of the expression of GLP1 receptor and related insulinotropic receptors through a mechanism that is dependent on peroxisome proliferator-activated receptor α (27, 28). Furthermore, 24-week combined treatment with exenatide and metformin was superior to exenatide and metformin monotherapies in improving menstrual cyclicity, ovulation rate, androgens, insulin sensitivity measures, and weight loss (11). On the contrary, the patients in our study did not experience statistically significant improvements in menstrual frequencies, or most of the hormones and components of the metabolic profile, most probably due to the short duration of the study. However, the COMBI arm proved the most successful among all three-treatment arms at reducing androstenedione. On the other hand, in a group treated with liraglutide monotherapy, there was a trend for increase in all observed androgens including androstenedione. This possible inferior effect of liraglutide monotherapy when compared with metformin alone or in combined regimen might result from metformin's unique beneficial direct effects on androgen production (29). Owing to the generally known methodological problem for assessment of androgens in PCOS, small sample size, and lack of any data on GLP1 effects on LH secretion, we cannot provide any firm conclusion about this observation. Furthermore, in our study, the COMBI arm was superior to both monotherapies in reducing glucose value after 120 min during OGTT. In addition, liraglutide, either alone or in combination with metformin, also seems to be more successful in the reduction of incidence of the IGT and the reversibility of fully blown metabolic syndrome when compared with MET monotherapy. Although the small sample size limits the generalizability of our results, we believe that GLP1 receptor agonist might be effective in preventing progression of prediabetes in patients with PCOS. Novel possible mediators and mechanisms need to be investigated to explain the effects of GLP1 agonists in obesity, IGT, type 2 diabetes, and PCOS. A possible role of a novel adipocyte-secreted hormone, named secreted frizzled-related protein 5 (SFRP5), has been shown to link obesity with diabetes in animal studies (30). Recently, in humans in a series of cross-sectional and interventional studies, it has been demonstrated that circulating SFRP5 is significantly lower in subjects with both IGT and newly diagnosed type 2 diabetes compared with subjects with normal glucose tolerance. Furthermore, overweight/obese subjects had significantly lower SFRP5 levels than lean individuals. Women with PCOS had significantly lower levels of SFRP5 than healthy women. Hyperglycemia decreased SFRP5 levels, whereas a 16-week intervention with liraglutide increased SFRP5 levels (31).

A prerequisite for every intervention, especially when applied to ameliorate a characteristic that is not associated with an immediate health threat such as obesity, is a very acceptable safety profile (21). In this study, the most frequent adverse events were nausea and diarrhea that were generally mild to moderate, subsided over time and did not correlate with weight loss. They were experienced by more than 50% of the subjects in LIRA and COMBI treatment arms. In the MET group, only one subject had temporary mild nausea and diarrhea. As it is known that GI side effects occur early during treatment with metformin and resolve over time, a significant differential effect between these two groups was probably partially associated with the study design including a pretreatment period of at least 6 months with metformin before randomization. Two (out of 40) patients on liraglutide dropped out because of GI side effects that were perfectly acceptable for all others. Hypoglycemic events were rare in all treatment arms. The s.c. injections do not seem to represent a major obstacle either.

We believe that a small drop out rate and better overall treatment satisfaction in our study, as opposed to a relatively large drop out rate in the study with exenatide (11), was conditioned by the choice of GLP1 receptor agonist. Liraglutide differs from exenatide in amino acid identity with human GLP1, clearance and pharmacokinetics, and frequency and timing of administration. It has been demonstrated in individuals with type 2 diabetes mellitus that liraglutide once a day caused less nausea, therapy discontinuation, hypoglycemia, and greater improvements in treatment satisfaction than exenatide twice a day (12). The short-term safety profile of both GLP1 receptor agonists in patients with type 2 diabetes, obese subjects without type 2 diabetes, and in women with PCOS seems to be acceptable. However, it is currently impossible to obtain precise estimates of the long-term risk of the more serious adverse effects such as pancreatitis or precancerous pancreatic lesions that have been claimed by some to be associated with GLP1-based therapies (21).

In summary, our study is the first to imply that obese women with PCOS who have not responded to standard weight loss strategies and metformin treatment might benefit from short-term intervention with liraglutide as an add-on therapy to metformin. We provide preliminary data that 12-week combined treatment with liraglutide and metformin was associated with significant weight loss and decrease in waist circumference in subjects who had previously been poor responders regarding weight reduction on metformin monotherapy. A short-term safety profile was acceptable. As weight has been recognized as the most modifiable baseline predictor of fertility in PCOS and its reduction instantly improved the likelihood of healthy pregnancy (32), we would speculate that after this intervention most benefits of this additional weight loss in poor responders to other treatments might translate in the improvement of their fertility. Larger trials of longer duration and post interventional follow-up are warranted to assess the efficacy and safety of combined LIRA–MET therapy in obese women with PCOS. The reproductive outcome after such intervention should be a focus of further investigation.

## Figures and Tables

**Table 1:** Characteristics (mean and s.d., *median, and interquartile range) at baseline and after 3 months of metformin or liraglutide or combined treatment.

	**MET** (*n*=14)		**LIRA** (*n*=11)		**COMBI** (*n*=11)		***P* values**
Pretreatment	Posttreatment (after 3 months)	Pretreatment	Posttreatment (after 3 months)	Pretreatment	Posttreatment (after 3 months)
Age (years)	31.3±9.4		31.5±6.4		31.1±5.1		NS
BMI (kg/m^2^)	36.6±3.5	36.1±3.8	39.3±4.2	37.9±4.0	37.6±5.1	35.3±5.5	T<0.001; C vs M<0.001
Weight (kg)	103.2±6.3	102±6.8	108.9±15.1	105.1±13.8	105.5±20.6	99±21.2	T<0.001; C vs M<0.001
Waist circumference (cm)	122.3±7.0	120.7±7.8	124.9±9.9	121.7±9.6	121.9±17.7	116.4±18.4	T=0.049; C vs M=0.050
BP systolic (mmHg)	121.9±13.1	116.8±14.6	123.3±15.4	114.9±12.6	125.0±13.0	126.7±18.2	NS
BP diastolic (mmHg)	74.1±12.2	70.2±9.4	77.2±15.6	71.8±9.9	80.0±6.6	79.9±7.8	NS
Total body % fat (%)	45.6±4.7	44.5±4.7	47.1±2.7	45.5±3.0	44.8±3.7	43.3±3.5	NS
VAT mass (g)	808.8±229.3	732.5±172.1	840.4±346.4	762.3±296.8	768.2±277.5	746.5±273.2	NS
VAT volume (cm^3^)	874.3±247.9	792.1±186.0	908.6±374.6	824.1±321.0	830.1±299.4	806.9±295.1	NS
VAT area (cm^2^)	167.7±47.6	151.9±35.6	174.3±71.9	158.1±61.6	159.4±57.6	154.9±56.8	NS
Menstrual cycles (*n*/month)	0.7±0.4	0.6±0.4	0.6±0.3	0.8±0.3	0.5±0.3	0.7±0.3	NS
HOMA-IR score	3.8 (3.8)*	2.5 (2.4)*	2.4 (2.3)*	2.1 (2.0)*	1.7 (4.9)*	2.1 (2.9)*	NS
Glu 0 min OGTT (mmol/l)	5.1 (0.9)*	5.5 (0.5)*	5.1 (1.2)*	4.7 (1.0)*	5.1 (0.2)*	4.6 (1.0)*	NS
Glu 120 min OGTT (mmol/l)	7.3±2.6	7.6±2.0	7.1±1.9	5.8±1.3	7.7±1.8	5.8±1.3	T=0.009; C vs M=0.006
Insulin 0 min OGTT (mU/l)	12.3 (13.3)*	12.7 (8.3)*	11.1 (9.6)*	9.4 (9.6)*	5.7 (11.8)*	9.4 (11.7)*	NS
Insulin 120 min OGTT (mU/l)	79.0 (112.1)*	86.9 (75.3)*	59.2 (97.0)*	68.4 (88.1)*	56.8 (73.2)*	55.8 (50.4)*	NS
Cholesterol (mmol/l)	4.7±0.6	4.6±0.4	4.9±0.7	4.7±0.7	5.3±1.1	4.6±0.8	NS
HDL cholesterol (mmol/l)	1.1±0.2	1.2±0.2	1.1±0.3	1.1±0.2	1.3±0.4	1.1±0.4	NS
LDL cholesterol (mmol/l)	2.9±0.6	2.9±0.5	3.2±0.8	3.1±0.5	3.4±0.8	2.9±0.8	NS
TG (mmol/l)	1.6 (0.5)*	1.3 (0.5)*	1.4 (1.1)*	1.1 (1.4)*	1.4 (0.4)*	1.4 (0.5)*	NS
Total testosterone (nmol/l)	1.7±1	1.5±0.9	1.8±1.1	2.2±1.4	2.1±1.3	1.6±0.7	NS
Free testosterone (pmol/l)	5.7±3.2	5±3.3	7.4±2.8	8±3.9	7.6±3.7	5.6±2.7	NS
SHBG (nmol/l)	20.0 (17.0)*	24.0 (11.0)*	16.0 (11.0)*	21.0 (12.5)*	36.5 (27.8)*	44.0 (31.0)*	NS
DHEAS (μmol/l)	6.2±3.2	5.8±3.6	4.3±2.2	4.6±3.2	5.7±2.1	5.3±2.4	NS
Androstenedione (nmol/l)	7.1±2.7	7.9±3.1	9±2.9	10.9±5.3	11.5±4.8	9.3±3.3	T=0.029; C vs L=0.030

For *P* values, T, overall effect after all treatments; M, MET; L, LIRA; C, COMBI; NS, not significant; BP, blood pressure; HOMA-IR, homeostasis model assessment of insulin resistance; Glu, glucose; TG, triglyceride; SHBG, sex hormone-binding globulin; VAT, visceral adipose tissue. Values of androgens in the healthy matched population: total testosterone, 0.3–3.5 nmol/l; free testosterone, 0.14–7.0 pmol/l; SHBG, 18–114 nmol/l; DHEAS, 3.6–12.9 μmol/l; androstenedione, 0.7–10.8 nmol/l.

## References

[bib1] Palomba S, Falbo A, Zullo F, Orio F (2009). Evidence-based and potential benefits of metformin in the polycystic ovary syndrome: a comprehensive review. Endocrine Reviews.

[bib2] Diamanti-Kandarakis E, Christakou CD, Kandaraki E, Economou FN (2010). Metformin: an old medication of new fashion: evolving new molecular mechanisms and clinical implications in polycystic ovary syndrome. European Journal of Endocrinology.

[bib3] Dunaif A (2008). Drug insight: insulin-sensitizing drugs in the treatment of polycystic ovary syndrome – a reappraisal. Nature Clinical Practice. Endocrinology & Metabolism.

[bib4] Pasquali R, Gambineri A (2009). Targeting insulin sensitivity in the treatment of polycystic ovary syndrome. Expert Opinion on Therapeutic Targets.

[bib5] Panidis D, Tziomalos K, Papadakis E, Kandaraki EA, Katsikis I (2013). The guidelines issued by the European Society for Human Reproduction and Embryology and the American Society for Reproductive Medicine regarding the induction of ovulation with metformin in patients with the polycystic ovary syndrome potentially require reconsideration. Hormones.

[bib6] Lord JM, Flight IHK, Norman RJ (2003). Metformin in polycystic ovary syndrome: systematic review and meta-analysis. BMJ.

[bib7] Harborne LR, Sattar N, Norman JE, Fleming R (2005). Metformin and weight loss in obese women with polycystic ovary syndrome: comparison of doses. Journal of Clinical Endocrinology and Metabolism.

[bib8] Abu-Hamdah R, Rabiee A, Meneilly GS, Shannon RP, Andersen DK, Elahi D (2009). Clinical review: The extrapancreatic effects of glucagon-like peptide-1 and related peptides. Journal of Clinical Endocrinology and Metabolism.

[bib9] Woods SC, D'Alessio DA (2008). Central control of body weight and appetite. Journal of Clinical Endocrinology and Metabolism.

[bib10] Vilsbøll T, Christensen M, Junker AE, Knop FK, Gluud LL (2012). Effects of glucagon-like peptide-1 receptor agonists on weight loss: systematic review and meta-analyses of randomized controlled trials. BMJ.

[bib11] Elkind-Hirsch K, Marrioneaux O, Bhushan M, Vernor D, Bhushan R (2008). Comparison of single and combined treatment with exenatide and metformin on menstrual cyclicity in overweight women with polycystic ovary syndrome. Journal of Clinical Endocrinology and Metabolism.

[bib12] Buse JB, Rosenstock J, Sesti G, Schmidt WE, Montanya E, Brett JH, Zychma M, Blonde L, LEAD-6 Study Group (2009). Liraglutide once a day versus exenatide twice a day for type 2 diabetes: a 26-week randomized, parallel-group, multinational, open-label trial (LEAD-6). Lancet.

[bib13] Zawadzki JK, Dunaif A, Dunaif A, Haseltine F, Merriam GR (1992). Diagnostic criteria for polycystic ovary syndrome. Polycystic Ovary Syndrome.

[bib14] Azziz R, Zacur HA (1989). 21-Hydroxylase deficiency in female hyperandrogenism: screening and diagnosis. Journal of Clinical Endocrinology and Metabolism.

[bib15] Ryo M, Maeda K, Onda T, Katashima M, Okumiya A, Nihida M, Yamaguchi T, Funahashi T, Matsuzawa Y, Nakamura T (2005). A new simple method for the measurement of visceral fat accumulation by bioelectrical impedance. Diabetes Care.

[bib16] DiBrezzo R, Fort IL, Brown B (1991). Relationships among strength, endurance, weight and body fat during three phases of the menstrual cycle. Journal of Sports Medicine and Physical Fitness.

[bib17] Genuth S, Alberti KG, Bennett P, Buse J, Defronzo R, Kahn R, Kitzmiller J, Knowler WC, Lebovitz H, Lernmark A (2003). Follow-up report on the diagnosis of diabetes mellitus. Diabetes Care.

[bib18] Matthews DR, Hosker JP, Rudenski AS, Naylor BA, Treacher DF, Turner RC (1985). Homeostasis model assessment: insulin resistance and β-cell function from fasting plasma glucose and insulin concentrations in man. Diabetologia.

[bib19] Hedblad B, Nilsson P, Janzon L, Berglund G (2000). Relation between insulin resistance and carotid intima-media thickness and stenosis in non-diabetic subjects. Results from a cross-sectional study in Malmo, Sweden. Diabetic Medicine.

[bib20] Nicklas BJ, Penninx BW, Ryan AS, Berman DM, Lynch NA, Dennis KE (2003). Visceral adipose tissue cutoffs associated with metabolic risk factors for coronary heart disease in women. Diabetes Care.

[bib21] Holst JJ, Deacon CF (2013). Is there a place for incretin therapies in obesity and prediabetes?. Trends in Endocrinology and Metabolism.

[bib22] Rosenstock J, Klaff LJ, Schwartz S, Northrup J, Holcombe JH, Wilhelm K, Trautmann M (2010). Effects of exenatide and lifestyle modification on body weight and glucose tolerance in obese subjects with and without prediabetes. Diabetes Care.

[bib23] Astrup A, Rössner S, Van Gaal L, Rissanen A, Niskanen L, Al Hakim M, Madsen J, Rasmussen MF, Lean ME, NN8022-1807 Study Group (2009). Effects of liraglutide in the treatment of obesity: a randomized, double-blind, placebo-controlled study. Lancet.

[bib24] Spanos N, Tziomalos K, Macut D, Koiou E, Kandaraki EA, Delkos D, Tsourdi E, Panidis D (2012). Adipokines, insulin resistance and hyperandrogenemia in obese patients with polycystic ovary syndrome: cross-sectional correlations and the effects of weight loss. Obesity Facts.

[bib25] Jayagopal V, Kilpatrick ES, Holding S, Jennings PE, Atkin SL (2005). Orlistat is as beneficial as metformin in the treatment of polycystic ovarian syndrome. Journal of Clinical Endocrinology and Metabolism.

[bib26] Viollet B, Guigas B, Sanz Garcia N, Leclerc J, Foretz M, Andreelli F (2012). Cellular and molecular mechanisms of metformin: an overview. Clinical Science.

[bib27] Maida A, Lamont BJ, Cao X, Drucker DJ (2011). Metformin regulates the incretin receptor axis via a pathway dependent on peroxisome proliferator-activated receptor-α in mice. Diabetologia.

[bib28] Mannucci E, Tesi F, Bardini G, Ognibene A, Petracca MG, Ciani S, Pezzatini A, Brogi M, Dicembrini I, Cremasco F (2004). Effects of metformin on glucagon-like peptide-1 levels in obese patients with and without type 2 diabetes. Diabetes, Nutrition & Metabolism.

[bib29] Johnson N (2011). Metformin is a reasonable first-line treatment option for non-obese women with infertility related to anovulatory polycystic ovary syndrome – a meta-analysis of randomized trials. Australian & New Zealand Journal of Obstetrics & Gynaecology.

[bib30] Ouchi N, Higuchi A, Ohashi K, Oshima Y, Gokce N, Shibata R, Akasaki Y, Shimono A, Walsh K (2010). Sfrp5 is an anti-inflammatory adipokine that modulates metabolic dysfunction in obesity. Science.

[bib31] Hu W, Li L, Yang M, Luo X, Ran W, Liu D, Xiong Z, Liu H, Yang G (2013). Circulating Sfrp5 is a signature of obesity-related metabolic disorders and is regulated by glucose and liraglutide in humans. Journal of Clinical Endocrinology and Metabolism.

[bib32] Legro RS (2012). Obesity and PCOS: implications for diagnosis and treatment. Seminars in Reproductive Medicine.

